# Dissecting the genetic basis of response to salmonid alphavirus in Atlantic salmon

**DOI:** 10.1186/s12864-025-11735-2

**Published:** 2025-07-11

**Authors:** Domniki Manousi, Dorota Monika Jaskula, Fabian Grammes, Tim Martin Knutsen, Shahmir Naseer, Samuel AM Martin, Thomas Moen, Marie Saitou, Sigbjørn Lien

**Affiliations:** 1 Centre for Integrative Genetics, Department of Animal and Aquacultural Sciences, Faculty of Biosciences, Norwegian University of Life Sciences, Oluf Thesens vei 6, Ås, 1433 Norway; 2 AquaGen AS, Havnegata 9, Trondheim, 7010 Norway; 3 Scottish Fish Immunology Research Centre, School of Biological Sciences, University of Aberdeen, Aberdeen, AB24 2TZ UK

**Keywords:** Atlantic salmon, Pancreas disease, Salmonid alphavirus, Structural variation, Whole genome duplication

## Abstract

**Background:**

The development of effective disease management strategies is crucial for the assurance of welfare and sustainability of the aquaculture industries. Pancreas disease (PD) is a major challenge faced by Atlantic salmon aquaculture with viral outbreaks resulting in substantial production losses and raising significant welfare concerns for farmed salmon populations. Previous research has identified several quantitative trait loci (QTL) associated with PD resistance accounting for a substantial additive genetic component. However, pinpointing the underlying causal variation remains challenging, partly due to the location of the QTL within duplicated regions of the Atlantic salmon genome that share high sequence similarity. The present study leverages the latest advancements in Atlantic salmon genomics in order to uncover the genetic landscape underlying PD resistance and identify genomic variation with putative functional impact on disease response.

**Results:**

Association mapping and haplotype analysis of fish challenged with salmonid alphavirus (SAV3), either through peritoneal injection or infectious cohabitation, confirmed the presence of a major QTL region on chromosome Ssa03. Additionally, another QTL on Ssa07 was detected, linked to infection-specific response. Transcriptomics analysis of the genes overlapping the Ssa03 QTL region revealed significant expression differences among three tandemly duplicated *gig1-like* genes, whereas allele-specific expression analysis detected several SNPs with putative functional impact on the particular genes. Use of long-read sequencing and construction of disease-associated haplotypes identified more complex variation in the region, offering a detailed exploration of the genetic architecture underlying PD resistance. Finally, integration of the regulatory landscape of Atlantic salmon during response to viral infection improved genomic resolution, providing novel insight into the potential causal variation underlying pancreas disease in Atlantic salmon.

**Conclusions:**

This study provides a detailed investigation of the genetic architecture underlying PD resistance in farmed Atlantic salmon. Using advanced genomic resources, three copies of the gig1-like gene were identified as likely causal candidates for a major QTL associated with PD resistance. Additionally, genomic variations with potential functional impact on gig1-like expression were uncovered. These findings hold promise for application in developing effective disease management strategies in Atlantic salmon aquaculture.

**Supplementary Information:**

The online version contains supplementary material available at 10.1186/s12864-025-11735-2.

## Background

In recent years, aquaculture has emerged as one of the fastest growing sectors of food production, contributing significantly to the global food supply chain [1]. With the rapid growth in aquaculture, several challenges have emerged regarding production sustainability and animal welfare, with one of the most significant problems being disease resistance.

Pancreas disease (PD), caused by salmonid alphavirus (SAV), poses a significant challenge for Atlantic salmon (*Salmo salar*) aquaculture. Although the SAV family consists of several subtypes (SAV1-SAV7), only SAV2 and SAV3 subtypes are found in Norway, with the SAV3 subtype being more aggressive in terms of mortality and clinical symptoms [2]. Infection with PD can occur during both the freshwater and seawater phases of salmon production and results primarily in inflammation, extensive necrosis and loss of exocrine pancreatic tissue. In addition to clinical symptoms, PD manifestations include loss of appetite, yellow mucoid gut contents, lethargy, abnormal swimming activity, skin erosion and skeletal myopathies [3, 4] while at the same time, immuno-compromised individuals become susceptible to secondary bacterial infections [5–7]. PD viral outbreaks result in mortality rates of up to 28% [8] and although recovery is possible, surviving individuals remain severely stunted, causing further damage to production [4]. PD virus is transmitted via water contact; viral particles from mucous and fecal excretions of infected or decomposing fish in the enclosure are released into the water, infecting healthy cohabitants [9]. Current efforts to mitigate the impact of PD include prophylactic vaccinations and selective breeding [10, 11]. Although application of such measures has managed to reduce the incidence and severity of PD outbreaks [11, 12], they remain only partially effective.

Due to the negative impact of PD on Atlantic salmon aquaculture, numerous studies have focused on uncovering the genomic basis of resistance to SAV3 virus. Genome scans using SNP-arrays have attributed a substantial genetic component to the disease with heritability ranging between 0.2 and 0.6 [13–15], while several quantitative trait loci (QTL) associated with PD resistance have been detected on chromosomes 3, 6, 7 and 23 (Ssa03, Ssa06, Ssa07 and Ssa23, respectively). Across studies, the QTL with highest significance for PD response is found on Ssa03, explaining between 15 and 41% of the total genetic variation [13–15]. However, this QTL covers a large region (approximately 27 Mb), making identification of candidate genes and putative causal variation particularly challenging.

A complicating factor for fine-mapping and identification of underlying causal variation is the physical position of the Ssa03 QTL; the particular QTL region is located at the end of the q-arm of Ssa03, sharing high sequence similarity with a duplicated region on the p-arm of Ssa06 [16]. The observed similarity is attributed to delayed rediploidization following the salmonid-specific whole-genome duplication event (Ss4R) that occurred approximately 89–125 million years ago [17]. The high sequence similarity between the two chromosomal regions resulted in a substantially fragmented assembly and poor resolution of the duplicated sequences in the first Atlantic salmon reference genome (ICSASG_v2; GCA_000233375.4) compromising downstream analyses [16].

Recently, some of these challenges have been addressed by the construction of a long-read based reference genome for Atlantic salmon (Ssal_v3.1; GCA_905237065.2), which has greatly improved contiguity and genomic resolution of complex genomic regions. In addition, functional information of the Atlantic salmon genome is currently available [18], providing invaluable insight over the regulation of genes during disease infection. The present study leveraged the latest advances in Atlantic salmon genomics to fine map the genomic loci associated with PD resistance and elucidate the underlying genomic variation landscape, providing novel insights for developing sustainable and effective disease management strategies.

## Materials and methods

### Resource populations

The present study utilized several populations originating from both commercial lines and from the breeding population of AquaGen AS. In particular, 293 fish from the AquaGen AS breeding population, hatched between 1998 and 2014, were used to provide whole-genome sequence level genotype information through short-read sequencing technologies. In addition to these samples, seven more individuals from the breeding population of AquaGen AS were used to provide whole-genome sequence level genomic information through long-read sequencing technologies.

Furthermore, four distinct populations (5,628 fish) from the AquaGen AS commercial line were used in this study, participating in independent *Salmonid alphavirus* subtype 3 (SAV3) viral challenge trials. The latter four populations, hatched during 2018, 2019, 2020 and 2022, were used in three distinct SAV3 survival challenges (year-classes 2018, 2019 and 2020) and one SAV3 viral cohabitation challenge trial (year-class 2022), respectively. Informed consent of participation for all privately owned individuals was provided by AquaGen AS.

**Fig. 1:**
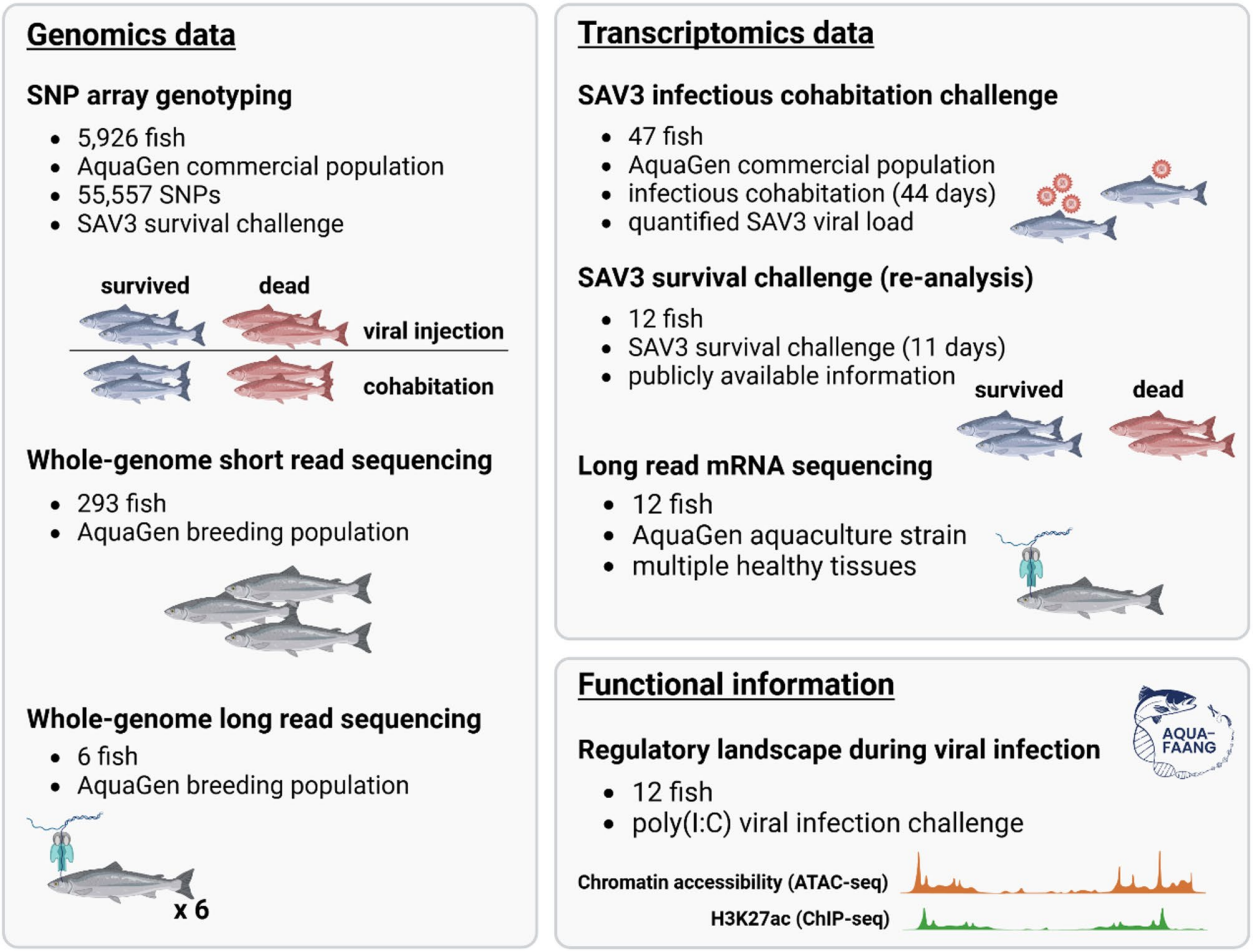
Visual representation of the material used in the present study. In brief, samples from several populations were used, originating from breeding and commercial lines of AquaGen AS. Origin of each population used in the study is marked in the figure while sampling and experimental details of the same groups are described in the respective methods sections. In addition to samples retrieved from commercial populations, transcriptomics (long read mRNA sequencing and gene expression data from a SAV3 survival challenge) as well as functional information were retrieved from previously published research. Further details regarding the origin of these data are provided in the respective methods section whereas informed consent for the use of these data was either not required (publicly available repositories) or provided by their rightful owners

Besides the populations described, the present study additionally leveraged transcriptomics and regulatory information from samples that participated in previously published research [15, 19, 20]. Details regarding the origin of these individuals can be found in their respective studies whereas informed consent for participation regarding these samples was either not required (publicly available data) or provided by their respective owners. A visual representation of the genomic material used in the present study is provided in Fig. 1.

### SAV3 survival challenge samples

Genotype and phenotype data from a cohort of 5,628 Atlantic salmon parr originating from three distinct AquaGen commercial populations (AquaGen AS), hatched between the years 2018, 2019 and 2020 were used in this study. Participating individuals were PIT-tagged and adipose fin clips were collected from anaesthetized fish for DNA extraction and SNP genotyping. SNP genotyping was performed using either of two proprietary AquaGen genotyping arrays (55,557 SNPs, Affymetrix axiom arrays Ssa70kv1 and Ssa70kv2, Table 1). Singular exception constituted the year class of 2020 for which genotype data were available for only a part of the challenge population (Table 1).

Each of the three year-groups participated independently in a controlled PD challenge trial at the VESO Vikan facilities (Namsos, Norway). Standard operating procedures were used for conduction of each SAV3 challenge, and all challenges were approved beforehand by the Norwegian National Animal Research Authority (FOTS) (approval no. 15233, 19332 and 23006 for conduction of the 2018, 2019 and 2020 challenge tests, respectively). For each challenge test, grouped samples of Atlantic salmon parr (3–4 g growth stage) were subjected to SAV3 infection either via peritoneal injection (IP group) or through cohabitation with injected fish (CH group). For the IP group, fish were first anaesthetized with 3 ml of 20% benzocaine in 10 L of water and then injected with 100 µl of a SAV3 isolate (Jnr 2618, passaged 3 times in CHSE-214 cell). Each challenge test was performed in a 0.6 m tank containing 125 L of 12 ºC water, with a 24:0 light - darkness regime and feeding according to appetite, whereas the ratio of CH to IP individuals per tank ranged between 1.0 and 1.4. Challenge tests spanned a duration of 7 to 9 weeks with tank inspections occurring two times a day. During inspection, dead and moribund fish were removed from the tank and individual survival as well as infection group of each fish were recorded accordingly. Fish found in a moribund state during the challenges as well as challenge survivors were first anesthetized and then euthanized using 6 ml of 20% benzocaine per 10 L of water. All infectious challenges were conducted in accordance with the legislation regarding experiments and procedures for live animals in Norway (Animal welfare Act of June 19th, 2009 and the Regulation on Animal Experimentation of January 15th, 1996).

### Whole-genome sequencing data and variant calling

A whole-genome level genotype dataset was generated using a collection of 293 whole genome resequenced (WGS) Atlantic salmon fish originating from the AquaGen breeding population (AquaGen AS). The particular population comprised of 94 ancestors hatched between the years 1998 and 2001 as well as a group of 199 fish hatched in 2014. The latter samples included direct ancestors (parents) of fish participating in the PD challenge trials of this study (year-class 2018). To produce genetic information from this population, fish were anaesthetized, and fin clips were obtained from each individual for DNA extraction and sequencing. DNA extraction followed standard protocols and whole-genome sequencing was performed using BGISEQ and Illumina sequencing technologies.

Produced DNA sequencing reads from all samples were aligned against the latest Atlantic salmon genome reference Ssal_v3.1 (GenBank accession GCA_905237065.2) using BWA-mem2 and duplicated reads were marked with Samblaster 0.1.26 [21, 22]. Following alignment, Samtools and the GATK4 tools ‘BaseRecalibratorSpark’ and ‘ApplyBQSRSpark’ were used in a custom - built pipeline in order to ensure high consistency of variant calling across samples sequenced using different sequencing platforms [23, 24]. Finally, genetic variants were called for each sample using the ‘DeepVariantWGS’ mode of the variant caller DeepVariant v1.1.2 and individually detected variants were merged across all fish using GL-NEXUS v1.4.1 [25]. Filtering of the GLNEXUS output followed previously described benchmark recommendations [26], omitting filtering for allele balance. Instead, the excess of heterozygosity was considered in this study, discarding variants with allele departures lower than 1e-8. Quality filtering of SNP variant calls from GL-NEXUS retained in total 16,339,880 biallelic SNPs.

### Filtering and imputation of whole genome sequencing variants in the SNP array genotyped fish

A reference population was created from the 293 WGS fish. Following previously established quality control protocols [27], SNP filtering was performed using PLINK2 [28] and the following threshold criteria: minor allele frequency (maf) < 0.01 and SNP calling rate < 0.90, retaining in total 6,115,911 SNPs. Prior to performing SNP imputation on the array-genotyped challenge populations, a cross–validation analysis was implemented in the WGS dataset in order to filter SNPs with high genotype inference accuracy. To achieve this, the WGS dataset was randomly divided into five subsets and the genotypes of each subset were sequentially masked, retaining only SNPs commonly found between the WGS samples and the SNP array-genotyped fish of the SAV3 viral challenges. Each masked dataset was then haplotyped and imputed back into WGS genotype density using Beagle 5.4 and the remaining four datasets as the reference [29, 30]. Imputed SNP genotypes were finally compared against the masked “true” genotypes and imputation accuracy - estimated as the r^2^ correlation between masked and inferred genotype - was assessed for each imputed SNP, discarding variants with r^2^ accuracy below 0.80. Filtering of SNPs with high imputation reliability in the WGS population retained a total of 891,641 SNPs.

For the SAV3 viral challenge, 5,628 fish were genotyped for 55,557 SNP using either of two Affymetrix SNP arrays (Table 1). SNP quality filtering with PLINK2 [28] removed variants with minor allele frequency < 0.02 and missing genotype call rate < 5%, retaining in total 53,295 SNPs. The reference and alternative alleles of filtered SNPs were then matched across those of the respective SNPs in the WGS population dataset and allelic consistency was corrected using the software conform-gt (version 24May16.cee.jar). The genotypes of allele - conforming SNPs on the SAV3 challenge fish were finally haplotyped using Beagle 5.4 and the genotypes of high imputation accuracy SNPs were inferred from the reference WGS dataset using Beagle 5.4 with customized haplotype and effective population options (overlap = 5, window = 39, and ne = 10000, respectively). To remove potential artefacts from the imputation process, the imputed dataset was additionally filtered against minor allele frequencies lower than 2% using PLINK2.

### Estimation of heritability and variance component analysis

SNP-based heritability for pancreas disease resistance, described as survival from a SAV3 infectious challenge (dead or alive), and survivability (survived days post infection, dpi) were estimated using GCTA [28, 31]. A genomic relationship matrix (GRM) was used, constructed using the imputed genotypes and the **GCTA-LDMS** method implemented in the same software. The particular method was used in order to account for linkage disequilibrium (LD) bias observed due to genotype inference and increased SNP density (whole-genome level) [32]. Heritability estimations followed the linear mixed model:

1 $$Y = X\beta + Zu + \in $$

where **Y** is a vector of ‘n’ records on PD resistance and/or survivability, **β** is a matrix of coefficients for the fixed effects as well as the intercept; **Z** is the corresponding incidence matrix and **u** is the additive genetic effect utilizing the GRM and is distributed as ~ N(0, Gσa^2^), where G corresponds to the GRM and σa^2^ is the additive variance. Finally, **e** is a vector of residual effects.

Data were analysed separately for each year-class as well as collectively across all challenge trials for PD resistance (death or survival) and infectious group (CH or IP). Depending on the type of collective analysis, infection type (IP and CH) and/or year-class were fitted as fixed effects. In addition, a series of bivariate analyses were run to estimate the genetic correlation between PD resistance and survivability for the collective datasets as well as for the independent year-classes and infection groups (Additional file 1: Supplementary table S1). To perform the bivariate analyses, a modification of model (1) was used where the **Y** is a matrix of n records on PD resistance and survivability.

### Genome-wide association study

A series of univariate genome-wide association analyses (GWA) were conducted to identify significant associations between PD resistance and viral infection approach (IP or CH) using the imputed SAV3 challenge fish. Association analysis of the challenge population used a linear mixed model approach, as this is implemented in the software BOLT-LMM [33]. The model considered individual year-class and viral infection group as covariates, as well as the first 5 genetic variance components (PCs) estimated from 53,295 array-genotyped SNPs using PLINK2 [28]. Association significance for each analysis was corrected for multiple testing-bias using the Bonferroni correction criterion implemented in RStudio [34]. In addition, the genome-wide significance cut-off threshold was estimated using the R package Rainbowr [35] and the uncorrected p-values. To calculate this cut-off threshold, the Bonferroni correction criterion was considered with a significance threshold of 0.05.

Finally, following the GWAS analysis, the approach described in Shim et al. was used to estimate the proportion of phenotypic variance explained by the SNP(s) with highest association for PD resistance [36]. Estimation used the information provided from the association analysis output in the following relationship:

2 $$\frac{{2{{\widehat {\beta \:}}^2}MAF(1 - MAF)}}{{2{{\widehat {\beta \:}}^2}MAF\left( {1 - MAF} \right) + {{\left( {se\left( {\widehat {\beta \:}} \right)} \right)}^2}2NMAF(1 - MAF)}}$$

where MAF notates the minor allele frequency for a given SNP, N is the sample size and se($\:\widehat{\beta\:}$) is the standard error of the effect size for that SNP.

### Haplotype block and linkage disequilibrium analysis

Haplotype-resolved (phased) genotypes of the imputed challenge population were used to define blocks of SNPs sharing substantial linkage disequilibrium (LD). Haplotype block analysis was carried out in PLINK v1.9 using the method of Gabriel et al. [37, 38]. To avoid possible disruption of LD patterns, the full length of PD associated chromosomes was analyzed. Haplotype analysis constructed 1,225 and 794 haplotype blocks for chromosomes Ssa03 and Ssa07, respectively. Within each of the two chromosomes, the haplotype block containing the highest associated SNP for PD resistance was retained.

In order to further refine the PD associated haplotypes, the LD patterns of SNPs inside the retained haplotype blocks were visualized in Haploview [37, 39]. For each block, the Pairwise LD relationships between each SNP and the QTL top-SNP were evaluated, filtering markers sharing near perfect LD (r^2^ > 0.95). Filtering was repeated until the retained SNPs of each block formed haplotype combinations with unique major and minor allele instances. Refined haplotype blocks were then converted into genotypes using the R package GHap [40, 41] and association significance was tested using Gemma [42]. For the haplotype-based association analysis, the same linear mixed model as before was used (model 1), together with the respective parameterization.

### Transcriptome analysis of a SAV3 infectious cohabitation challenge

Transcriptomics data were obtained from an AquaGen AS commercial population, hatched in 2022, that participated in a SAV3 infectious cohabitation challenge. For the infectious cohabitation challenge, 618 healthy and PIT-tagged parr (average weight 17.3 g) with known genotype information for 63,849 SNPs (AquaGen proprietary Affymetrix 70kv3 SNP genotyping array) were placed in a common garden experiment together with SAV3 infected fish from the same aquaculture strain. Infected individuals were first anaesthetized and then virally injected with SAV3 (Jnr 2618, passaged 3 times in CHSE-214 cells, 100 µl inoculation volume). After 44 days of viral cohabitation all fish were anaesthetized and euthanized using 6 ml of 20% benzocaine per 10 L of water, and heart tissue of infected cohabitants was sampled and stored in RNAlater (Thermofisher). Similarly to the SAV3 survival challenge tests, conduction of the viral cohabitation challenge was approved beforehand by the Norwegian Animal Research Authority (approval no. 29261) and followed standard operating procedures of VESO Vikan (Namsos, Norway).

Total RNA extraction as well as qPCR for SAV3 viral load was conducted by BlueAnalytics (Bergen, Norway). Selection of samples for mRNA sequencing was based on the individual genotype for the Ssa03 QTL top-SNP (Ssa03_95086730) and infection severity– classified as the quantified viral load title (difference between qPCR viral titre and qPCR expression of housekeeping gene *elf1a*). Extracted mRNA material from 47 selected samples (18 reference homozygotes, 10 heterozygotes and 19 alternative homozygotes) with viral load values (delta Ct = Ct SAV– Ct EF1α) ranging between 1 and 8 was sequenced, producing approximately 150 million pair-end reads per individual. Raw RNA sequencing reads were then quality filtered using fastp v0.23.2 [43] to remove sequencing adapters and low quality reads and were then aligned against the Atlantic salmon transcriptome (Ssal_v3.1, accession number GCA_905237065.2) using STAR v2.7.10 [44]. Gene expression of aligned reads was quantified using FeatureCounts v2.0.3 [45], whereas sequencing reads that did not map successfully to the salmonid genome where aligned against the SAV3 viral genome using Kraken2 v2.1.2 [46] to verify correspondence between viral concentration and qPCR-based viral load (p-value < 2.2e-16, spearman’s rank test).

In order to investigate differences in expression profile between mildly and severely infected samples, quantified expression was used to perform a differential gene expression (DEG) analysis using the package DESeq2 in RStudio [34, 47, 48]. To reduce the dimensionality of the viral load dataset (titre scaled between 1 and 8), quantified viral load was classified as either high, medium, or low (values < 4 = high viral load, values > 6 = low viral load, in-between values = medium viral load). To observe higher contrasts in gene expression profile, the DEG analysis focused exclusively on comparison between samples grouped into high and low viral load classes, each of which consisted of 12 samples. Genes with significant expression differences were filtered based on False discovery rate (FDR) adjusted p-value (p-value < 0.05) and visualization of expression patterns was performed using the package ‘ggplot’ in Rstudio [49].

### Transcriptome re-analysis of a SAV3 viral injection challenge

Publicly available mRNA sequencing data from a SAV3 viral challenge [17] were obtained from the European Nucleotide Archive repository (Study accession: PRJNA590166). In this challenge, 12 samples were peritoneally (IP) injected with SAV3 virus and participated in a survival challenge for four weeks. During the experiment, any participant fish found in moribund state was removed and survival was recorded as either dead or alive after 11 days.

To produce comparable results between experimental setups, quality filtering and quantification of the survival challenge transcriptomics data followed the same pipeline as previously described, omitting the step of viral sequence quantification. Similarly to the viral cohabitation samples, a DEG analysis was performed using DESeq2 to explore differential gene expression between IP fish that survived or died after 11 days of the viral challenge and genes with (FDR) adjusted p-value < 0.05 were retained as significantly differentially expressed [47].

### Allele specific expression (ASE) analysis using infectious cohabitation samples

An allele specific expression (ASE) analysis was performed in the 10 SAV3 infected cohabitants that carried the heterozygote genotype for the Ssa03 QTL top-SNP (ssa03_95086730) in order to identify SNP variants with allele specific impact on gene expression. For the ASE analysis, all biallelic SNPs overlapping the Ssa03 QTL region (Ssa03: 95,054,413–95,127,543) were obtained from the quality filtered WGS dataset - irrespective of imputation accuracy score. Retained SNPs were haplotyped using Beagle v5.4 [29] and the LD relationship between each of these SNPs and the QTL top-SNP was estimated using VCFtools v0.1.16 [50]. SNPs with LD score higher than 0.6 were then re-coded as heterozygous and used to identify mRNA sequencing reads mapping uniquely within the Ssa03 QTL region. Unique read mapping was performed using STAR v2.7.10 aligner and the option *--waspOutputMode* [16, 44]. Furthermore, in order to account for increased sequence similarity due to the salmonid-specific genome duplication [51], the alignment process was adjusted using the option *–outSAMmapqUnique*. Allele specific expression was estimated for each SNP based on the uniquely mapping read alignment using the GATK4 tool ASEReadCounter [52]. Finally, SNP-wise significance between gene expression and the alleles of each SNP was estimated using the exact binomial test option ‘*binom.test’* in R [34, 48].

### Detection of complex variants using long-read sequencing technologies

To analyze complex variation in the salmonid genome, whole-genome sequencing information was obtained from six fish originating from an AquaGen breeding population (AquaGen AS). The fish were anesthetized using a 20% benzocaine overdose bath and then bludgeoned, and blood was sampled from the bludgeoning point for DNA extraction. Conduction of the sampling took place at an AquaGen AS licensed facility (Steigen, Norway), following the legislations regarding animal welfare and operation of aquaculture facilities in Norway (Animal welfare Act of June 19th, 2009, and regulation on the Operation of Aquaculture Facilities of June 17th, 2008).

Extraction of DNA for these samples followed standard protocols and genomic material was sequenced using Oxford Nanopore (ONT) sequencing technologies on the PromethION sequencing platform. In total, DNA sequencing reads were produced for each sample, providing a sequencing coverage of approximately 20x for each sequenced individual. In addition to the obtained fish, the present study also included publicly available sequencing information from the individual used to construct the Ssal_v3.1 genome assembly (sample accession SAMEA8062739). Sequencing quality and therefore coverage of the particular individual was significantly higher (approximately 70x).

Sequencing reads for the seven samples were quality filtered using Filtlong v0.2.1 to remove the worst 10% of reads as well as reads with length shorter than 4000 bp. Filtered output was then aligned against the Atlantic salmon genome reference Ssal_v3.1 using minimap2 [53, 54] and reads overlapping up to 100 Kb upstream and downstream of the Ssa03 QTL region (Ssa03: 94,976,092–95,221,176) were extracted using Samtools v1.11 [23]. Sequencing read-based haplotyping was performed on the aligned sequence of each sample using the software PEPPER to link adjacent variants through Recurrent and Convolutional Neural Networks. Haplotyped reads were then tagged to a respective haplotype using the software Margin, implemented within the PEPPER-Margin-DeepVariant r0.8 workframe [55]. Haplotype-tagged reads were classified as either of two haplotypes for reads with high confidence discrimination, whereas reads with low confidence on haplotype discrimination were tagged as ambiguous. Based on the assigned tags, haplotype resolved as well as “ambiguous” reads were filtered for each of the two haplotypes of each sample to ensure sufficient depth for homozygous individuals as well as for homozygous regions of heterozygous individuals. Finally, assemblies of each haplotype were produced using the option *--nano-raw* in Flye v2.9 [56]. Each of the assembled haplotypes consisted of singular contigs with read coverage ranging between 7 and 17. Variant calling and genotyping of the 14 assembled haplotypes was performed using the pangenome graph-based tool Minigraph-Cactus v2.6.9 [57]. Implementation of the software followed user guidelines with the exception of the *‘graphmap-split’* computational step. To distinguish disease associated haplotypes in the long-read sequenced fish, the allele segregation of SNP variants matching the Ssa03 QTL top-SNP as well as the set of 10 SNPs sharing LD higher than 0.95 in the SAV3 challenge data was assessed. Finally, variants with distinct allele segregation patterns between disease-associated haplotypes that carried either the reference or alternative top-SNP alleles were retained (Additional file 1: Supplementary Table S4).

### Long-read sequencing transcriptomics data and manual annotation of the Ssa03 QTL genes

Whole genome transcriptomics data from ONT long read sequencing technologies were obtained in order to study the structure of PD associated genes [20]. The dataset included sequencing samples from multiple tissues (brain, liver, head kidney, muscle, gill, intestine, and gonad) of 12 healthy Atlantic salmon individuals that participated in the AQUA-FAANG project (https://www.aqua-faang.eu/). DNA extraction and sequencing details for the respective samples have been previously described [19]. In brief, quality filtered sequencing mRNA reads of all sample tissues were aligned against the latest Atlantic salmon genome reference (Ssal_v3.1, accession number GCA_905237065.2) using minimap2 [53, 54] and gene expression was quantified using the Flair bioinformatics pipeline [58].

Aligned transcriptomic information from the long read sequencing data was inspected manually using the Integrative Genomics Viewer platform [59] and the distribution of mRNA sequencing reads was compared against short-read transcriptomics data of samples infected with high viral load in the previously described SAV3 infectious cohabitation challenge. In addition, two publicly available versions of the Atlantic salmon transcriptome (Ssal_v3.1, accession numbers: GCA_905237065.2 and GCF_905237065.1) were also consulted in order to assess the structure of genes associated with PD resistance (Fig. 7). The nucleotide sequence corresponding to the short and long mRNA read alignment was extracted and coding sequence for each likely exon region was predicted using the open reading frame (ORF) tool, ORF Finder that is implemented in the NCBI platform [60] (Additional file 1: Supplementary table S3). Prediction was performed using default settings (minimum ORF > 75 bp and only matching an ATG start codon). Finally, the multiple amino acid sequence alignment of predicted exons was used to perform protein domain prediction using the InterPro database with default settings [61].

### Investigation of variants with putative regulatory impact

In order to identify genomic variation with putative regulatory impact on the Ssa03 QTL region, the regulatory landscape of Atlantic salmon during viral infection stimulation was investigated. Regulatory information regarding histone modification (ChIP-seq) and chromatin accessibility (ATAC-seq) were obtained from an in vivo experiment of artificial Interferon (IFN) stimulation of salmon parr head kidney (HK) tissue using the viral mimic poly(I:C). The provided data were part of the Aqua-FAANG ImmunoMap project (AQUA-FAANG consortium, https://www.aqua-faang.eu/) and a detailed explanation regarding DNA extraction and production of ChIP-seq and ATAC-seq signal peak data is provided elsewhere (protocols are available at https://data.faang.org/home, under the University of Aberdeen submission of 2021). Briefly, H3K27ac and H3K27me3 sequencing data from ChIP-seq and chromatin accessibility data from ATAC-seq were obtained from six samples treated to 100 µg of poly(I:C) and six samples treated to control (PBS media) peritoneal injections [18]. Sequencing reads were quality filtered and processed using the nf-core pipelines ChIP- and ATAC-seq, respectively to produce quantified signal peaks [62, 63]. Detection of peaks with significant differences in signal abundance between IFN stimulated and control samples was performed using DESeq2 in Rstudio and intervals of significant difference in signal abundance were filtered based on FDR adjusted p-value < 0.05 [47, 63].

### Phylogenetic analyses of PD associated *gig1-like* genes

The manually annotated amino acid sequence for the three *gig1-like* gene copies was used to perform a protein BLAST search on Ensembl (release version 112) and NCBI public databases in order to identify *gig1-like* gene copies on chromosome Ssa06 in Atlantic salmon [64, 65]. Genes located within ohnolog regions of the Atlantic salmon genome were filtered based on synteny analysis information obtained through the Salmobase platform (https://salmobase.org). Synteny filtering retained the gene ENSSSAG00000003892 (Ssa06:5,558,971-5,559,951). The amino acid sequence for that gene was retrieved and aligned against the three Ssa03 gene copies using MAFFT v7.511 [66].

In order to investigate the life history of the four Atlantic salmon *gig1-like* genes, an additional protein BLAST was performed to detect homologous genes with high similarity in the Northern pike (*Esox lucius*), a close ancestral species that did not undergo the salmonid-specific whole genome duplication. The coding sequence of the four *gig1-like* salmonid genes together with the Northern pike orthologous gene showing highest amino acid sequence similarity (ENSELUG00000010316, 11:36,088,635 − 36,091,358) were aligned using MAFFT v7.511 [66]. Multiple sequence alignment used default options and the automated model selection strategy in order to utilize the best fitting model for the provided dataset. Furthermore, a neighbor joining tree was constructed based on the gap-free and moderately conserved sites of the coding sequence alignment and bootstrap values were calculated using 500 replicates. The produced phylogenetic tree was visualized using the iTOL platform [67].

## Results

### Pancreas disease SAV3 challenge trial

**Fig. 2:**
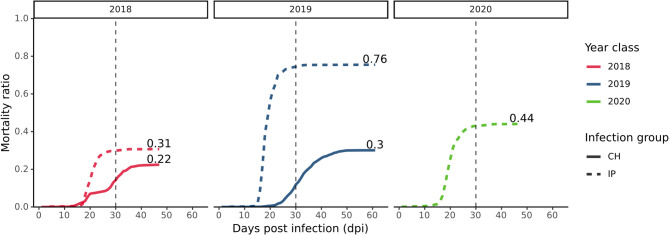
Mortality curves of three independent survival challenges of fish infected with Salmonid Alphavirus (SAV3), classified by the year they were carried out. In total 5,628 individuals were infected either by peritoneal SAV3 injection (IP group: 1,112, 1,023 and 1,060 fish of year class 2018, 2019 and 2020 respectively) or by infectious cohabitation with virally injected individuals (CH group: 1,137 and 1,296 fish of year class 2018 and 2019, respectively). Colors indicate the challenge year class while dashed and solid lines represent the IP and CH infection groups, respectively. Numbers above each line indicate the mortality rate at termination of each trial, 48 dpi, 61 dpi and 47 dpi for the challenges of year class 2018, 2019 and 2020, respectively

Our analysis utilized genotype and disease survival data collected from three independent challenge trials of Atlantic salmon fish infection with the Salmonid alphavirus SAV3 (Table 1; Fig. 2, Materials and methods). The three challenges were carried out between 2018 and 2020, classifying the participating fish respectively. For each of the three trials, fish were infected with Salmonid alphavirus (SAV3) either via peritoneal viral injection (IP infection group: 1,112, 1,023 and 1,060 fish of year class 2018, 2019 and 2020 respectively) or through infectious cohabitation with injected individuals (CH infection group: 1,137 and 1,296 fish of year class 2018 and 2019, respectively), with the latter method mimicking SAV3 transmission during a natural aquaculture outbreak scenario. Mortality rates reported during each trial showed a uniform and sharp increase at approximately 15 days post infection (dpi) for IP infected fish and at approximately 25 dpi for the CH group (Fig. 2). Similarly, mortalities across the three challenges reached a uniform plateau at approximately 30 and 45 dpi for IP and CH infected fish, respectively (Fig. 2). By termination of the three trials, the mortality rate of IP infected fish was moderate to high (31–76%), whereas mortality of infected cohabitants (CH) was substantially lower (22–30%) (Table 1 and Fig. 2). Estimated heritability of PD resistance estimated across all challenges was moderately high (0.28 +/- 0.08), whereas infection-based heritability was higher for IP infected fish (0.36 +/- 0.02) and considerably lower for the CH infection group (0.25 +/- 0.03). Lastly, disease survival (dead/alive) and survivability (dpi) were highly correlated across challenges (~ 99%, Additional file 1: Supplementary table S1) and therefore the following association analyses focused exclusively on the survival phenotype of PD resistance. Heritability and genetic correlation estimates of PD resistance and survivability for each year class and infection group are provided in Supplementary table S1 (Additional file 1).

**Table 1:** Composition of the PD study population. In total 5,628 fish were used in the present study that originated from three distinct populations hatched between the years 2018, 2019 and 2020. Individuals were genotyped using proprietary AquaGen genotyping arrays prior to the infection challenge. Infection of participating individuals occurred either through peritoneal injection (IP group) or via cohabitation of healthy fish together with injected individuals (CH group). Singular exception constitutes the 2020 year-class where only IP infected individuals were available for the analysis

Year class	IP group	CH group	Genotyping array	Challenge termination (days post infection)	Mortality rate (CH/IP group)	Total
**2018**	1,112	1,137	Ssa70kv1	48	22% / 31%	2,249
**2019**	1,023	1,296	Ssa70kv2	61	30% / 76%	2,319
**2020**	1,060	-	Ssa70kv2	47	- / 44%	1,060
**Total**	3,195	2,433				5,628

### Two QTL regions govern response to PD

To enhance the mapping resolution of QTL related to PD resistance in Atlantic salmon, we improved the genome scan by imputing missing genotypes in the PD challenge population and conducted a genome-wide association study (GWAS) using the inferred dataset. More specifically, the application of genotype imputation inferred genotypes for 885,287 missing SNPs, ultimately increasing the genotype density of the PD challenge population to 938,582 SNPs.

**Fig. 3:**
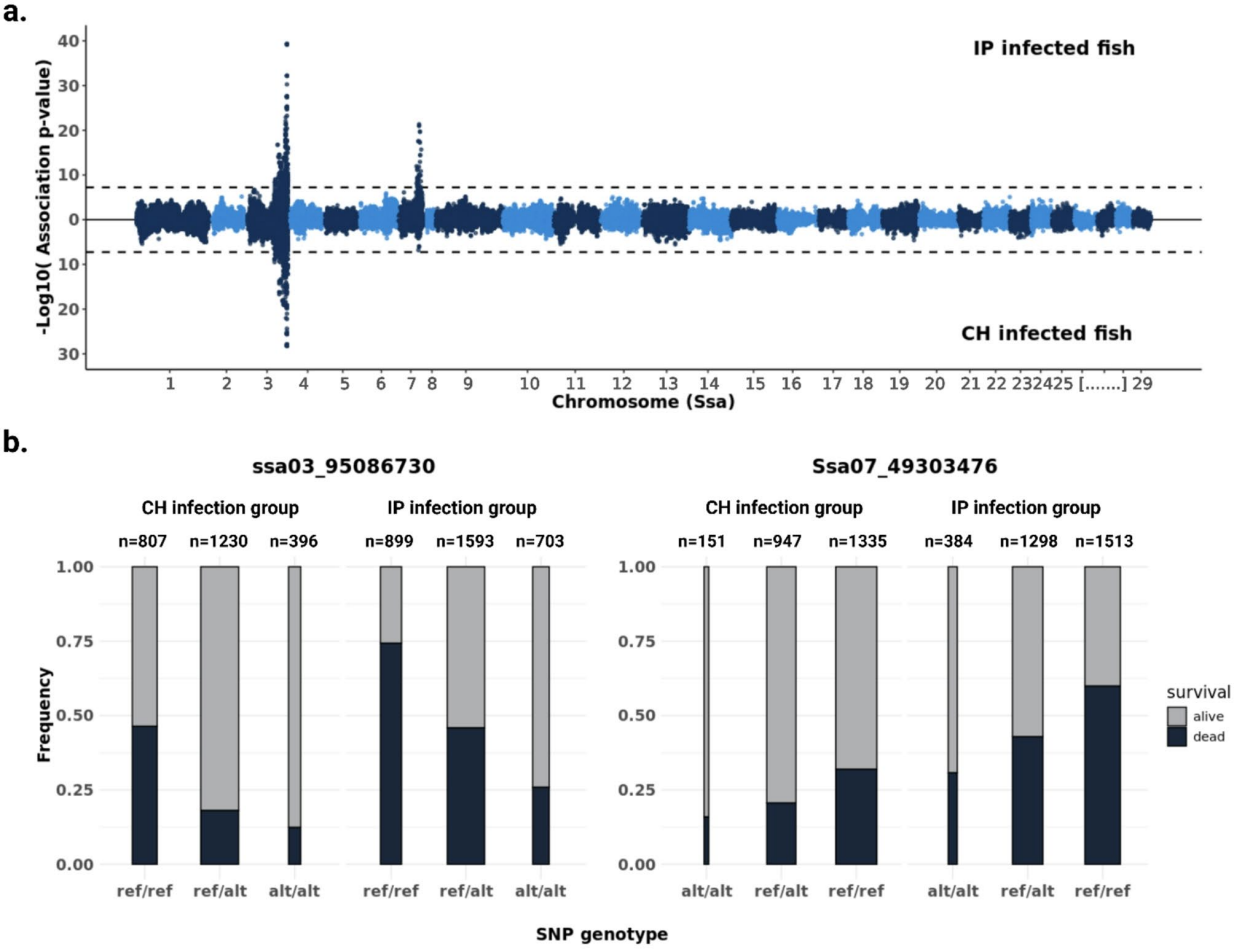
(**a.**) Genome wide association analysis for resistance against PD using SAV3 challenge populations of virally injected (IP) fish and infected cohabitants (CH). Association significance is adjusted for multiple testing bias using the Bonferroni correction criterion while dashed lines indicate the genome wide significance threshold. (**b.**) Genotype frequencies of the two SNPs with highest association to PD resistance for in the QTL regions on Ssa03 (left) and Ssa07 (right). Width of bars is adjusted to the number of fish with the respective Reference (ref/ref) or alternative (alt/alt) homozygote and heterozygote (ref/alt) genotype for the Ssa03 and Ssa07 QTL top-SNPs, respectively

Association testing and correction for multiple testing bias (p_Discovery_ < 10^− 7.23^) detected significant results for PD resistance on chromosomes Ssa03 and Ssa07 (Fig. 3). The most significant association peak was located within a broad region (33 Mb) on Ssa03 (Ssa03: 64,745,587–97,690,777 Mb), while a second peak appeared within an 11 Mb region on Ssa07 (Ssa07: 43,093,416–54,519,108). Within each QTL region, the highest association signals were detected for the SNPs ssa03_95086730 and ssa07_49303476, explaining 4% and 2% the overall phenotypic variance, respectively. Interestingly, independent analyses of PD survival for each of the two viral infection groups (IP or CH fish) both detected the QTL on Ssa03 however, the QTL on Ssa07 was significantly associated only in the IP infection group (Fig. 3).

**Fig. 4:**
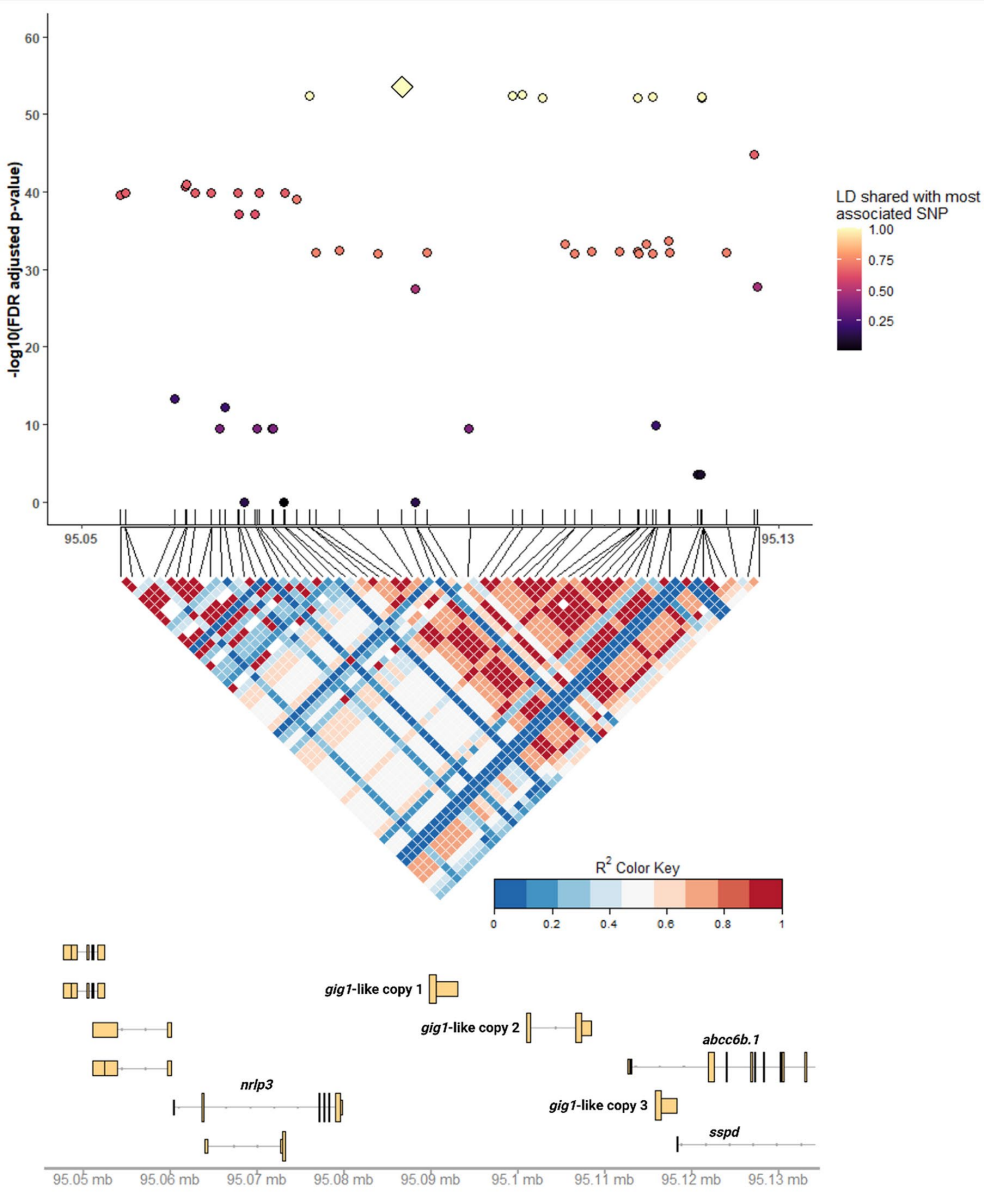
Linkage disequilibrium and haplotype block analyses within the Ssa03 QTL region. Each colored point represents an individual SNP within the Ssa03 QTL region for PD (x-axis) with statistical association to PD resistance (y-axis), while the large diamond shape indicates the SNP with highest association, hereby referred to as the top-SNP. The color for each point represents the linkage disequilibrium (LD) shared between each SNP and the top-SNP of the Ssa03 QTL, while the LD plot below highlights a narrow genomic region of high LD. Underneath, the gene landscape overlapping the QTL region is shown, based on the Ensembl functional annotation (release 112). In particular, the narrow QTL region overlapped 6 genes, namely *nlrp3*, three copies of *gig1-like*, *abcc6.b* and *sspd*

Building on the GWAS results for PD survival, we further investigated the QTL regions on Ssa03 and Ssa07 and performed linkage disequilibrium (LD) analyses to identify narrow genomic regions containing haplotypes with SNP alleles most strongly associated with PD resistance. Analyses for the Ssa03 QTL region revealed a narrow LD block in position Ssa03:95,054,413–95,127,543 consisting of 58 SNPs. Of these, 10 SNPs were found in almost perfect LD with the most significantly associated variant from the GWAS, narrowing the most likely candidate QTL region to 45 Kb (Ssa03:95,076,092–95,121,176). Using the Ensembl gene annotation (release v112), we detected six genes overlapping the particular region (Table 2). Since only one of the six genes was annotated with a gene name, a cross-search against publicly available databases was conducted. This identified the genes as ‘NACHT, LRR and PYD domains-containing protein 3-like’ (*nlrp3)*, three copies of a gene previously characterized as *gig1* [20] and *abcc6b.1*, respectively. No matching hit was available for the sixth identified gene (ENSSSAG00000108304), thereafter referred to as *sspd* (Fig. 4 and Table 2).

Similar investigation of the Ssa07 QTL region revealed a 23.6Kb haplotype block (Ssa07:49,279,835–49,303,476) consisting of 18 SNPs. Selection of SNPs in almost perfect LD with the highest associated variant further narrowed the candidate region to 23.1 Kb (Ssa07:49,280,373–49,303,476), overlapping the two genes *scaf11 and slc38a2* (ENSSSAG00000015784 and ENSSSAG00000015905, respectively, Table 2 and Additional file 2: Supplementary Fig. 1).

Analysis of allele combinations within the haplotype blocks revealed in total 16 distinct haplotypes in the SAV3 challenge population (Additional file 1: Supplementary Table S2). Application of a cutoff threshold (frequency > 0.2) retained two haplotypes for each QTL block. Association testing using the selected haplotypes and the same PD survival population as before yielded highly significant results (GWAS p-value < e-17). Despite the significant genotype and haplotype association results for both QTLs on Ssa03 and Ssa07, lack of significant association from independent analysis of the IP and CH infection groups revealed that only the Ssa03 QTL was significantly associated with PD resistance irrespective of infectious route. Based on these findings, subsequent analyses focused primarily on the narrowed QTL region on Ssa03.

**Table 2:** Genes overlapping the regions associated with PD response. Haplotype analyses revealed two narrow regions with significant association to PD resistance on chromosomes Ssa03 and Ssa07. The two regions were overlapping six and two genes, respectively. The physical coordinates and name of each gene is shown in the table, together with the respective gene accession number based on the Ssal_v3.1 ensembl and NCBI public annotations

Chromosome	Location	Gene name	Gene accession (Ensembl)	Gene accession (NCBI)
Ssa03	95,060,336 − 95,079,624	*NACHT*,* LRR and PYD domains-containing protein 3-like*	ENSSSAG00000071002	LOC106601922
Ssa03	95,085,053–95,090,404	*Gig1-like copy 1*	ENSSSAG00000119632	LOC106596079
Ssa03	95,100,560 − 95,107,946	*Gig1-like copy 2*	ENSSSAG00000094181	LOC106602070
Ssa03	95,115,341 − 95,117,818	*Gig1-like copy 3*	ENSSSAG00000121001	LOC106602018
Ssa03	95,112,232 − 95,187,058	*abcc6b.1*	ENSSSAG00000044528	LOC106601939
Ssa03	95,117,752 − 95,146,420	*sspd*	ENSSSAG00000108304	
Ssa07	49,266,424 − 49,292,752	*SCAF11*	ENSSSAG00000015784	LOC106609516
Ssa07	49,301,132 − 49,313,096	*slc38a2*	ENSSSAG00000015905	LOC106609517

### Transcriptome analyses and functional annotation highlight the importance of the gig genes within Ssa03 QTL region

To identify genes responsive to PD virus, we analysed gene expression data from two different experiments involving SAV3 viral infection. First, we studied expression patterns in PD infected fish with high or low viral concentration titre (viral load) following an infectious SAV3 cohabitation (CH) challenge. Differential gene expression analysis identified 14,712 genes with significant differences in expression profile between fish with high and low viral load (FDR adjusted p-value < 0.05). Of these, 7,225 genes were significantly upregulated in fish with low viral titre, whereas 7,487 genes were downregulated. Secondly, we reanalysed gene expression data from a previous study [15] that used SAV3 virally injected (IP) fish with survival records (dead or alive) following an four-week PD infectious challenge. This analysis identified 18,760 genes with significant expression differences between survivors and moribund fish in the four-week infectious trial. Of these, 10,024 genes were up-regulated in IP survivors, while 8,736 genes were down-regulated. Within the Ssa03 QTL region, both experiments revealed significant genome-wide expression differences for three *gig1-like* genes in response to SAV3 infection, indicating that these genes are likely candidates underlying the Ssa03 QTL (Fig. 4B and C).

**Fig. 5:**
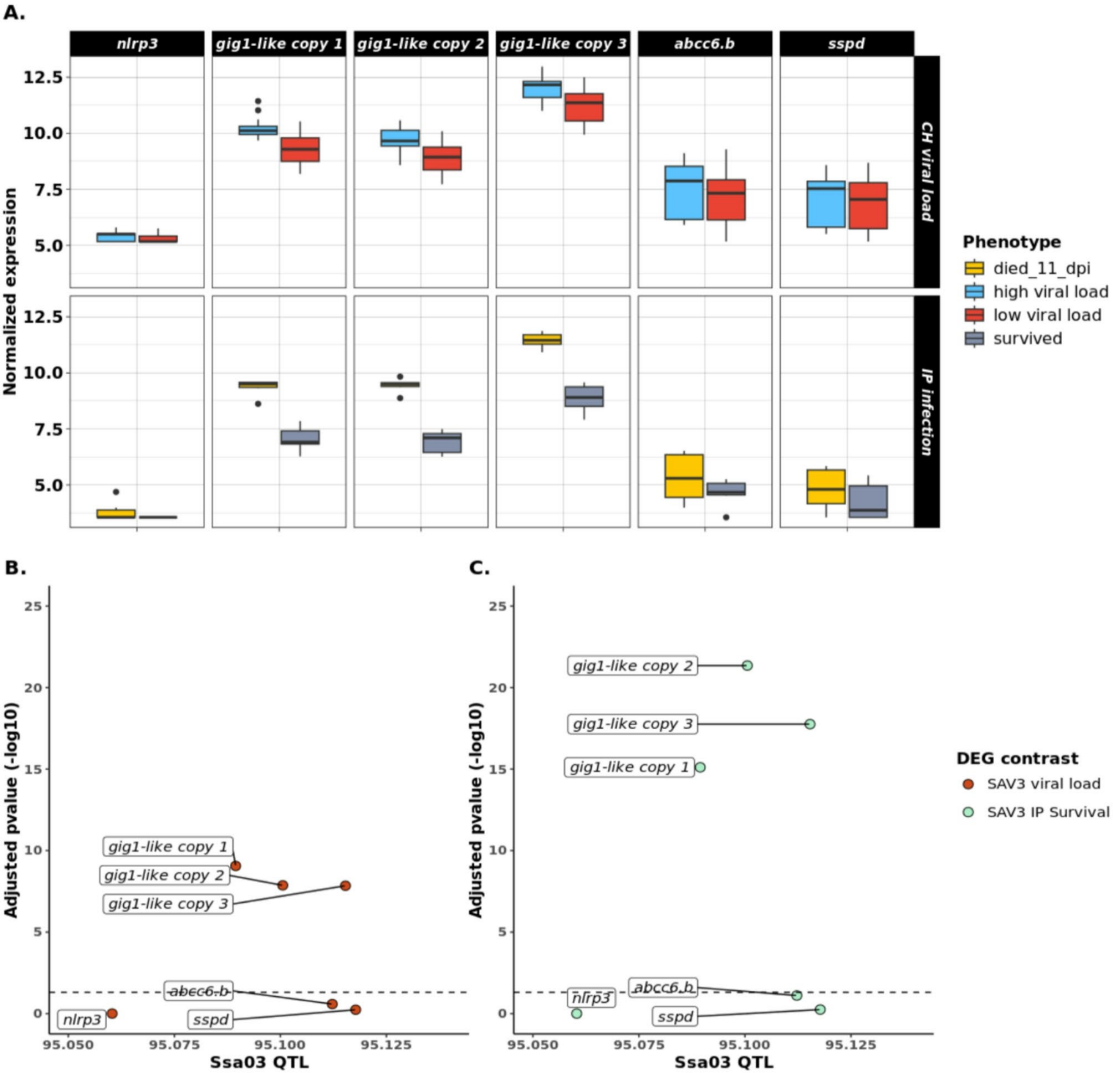
**(A)** Differences in expression pattern of genes overlapping the Ssa03 QTL region across two SAV3 infectious challenge studies. Top bins show gene expression of samples infected with high or low viral load during a viral cohabitation (CH) challenge. Bottom bins show gene expression of SAV3 virally injected samples (IP) that survived or died after 11 days of a four-week survival challenge trial. X-axis represents the expression of each gene with colors indicating the respective phenotypes for each of the two studies. Y-axis shows the expression level of genes represented as the log2-normalized value of mapped reads for each gene. **(B)** Differential gene expression analysis focusing on low/high viral load in CH infectious challenge individuals within Ssa03 QTL haplotype block. The physical position of genes is noted on x-axis whereas the y-axis represents the statistical significance of differential gene expression, expressed as the -log10 of the FDR adjusted p-value, with higher values indicating greater significance. Dashed lines represent the 0.05 FDR adjusted p-value cutoff point. **(C)** Differential gene expression analysis focusing on death/survival in IP survival challenge individuals within Ssa03 QTL haplotype block. The physical position of genes is noted on x-axis whereas the y-axis represents the statistical significance of differential gene expression, expressed as the -log10 of the FDR adjusted p-value, with higher values indicating greater significance. Dashed lines represent the 0.05 FDR adjusted p-value cutoff point

**Fig. 6:**
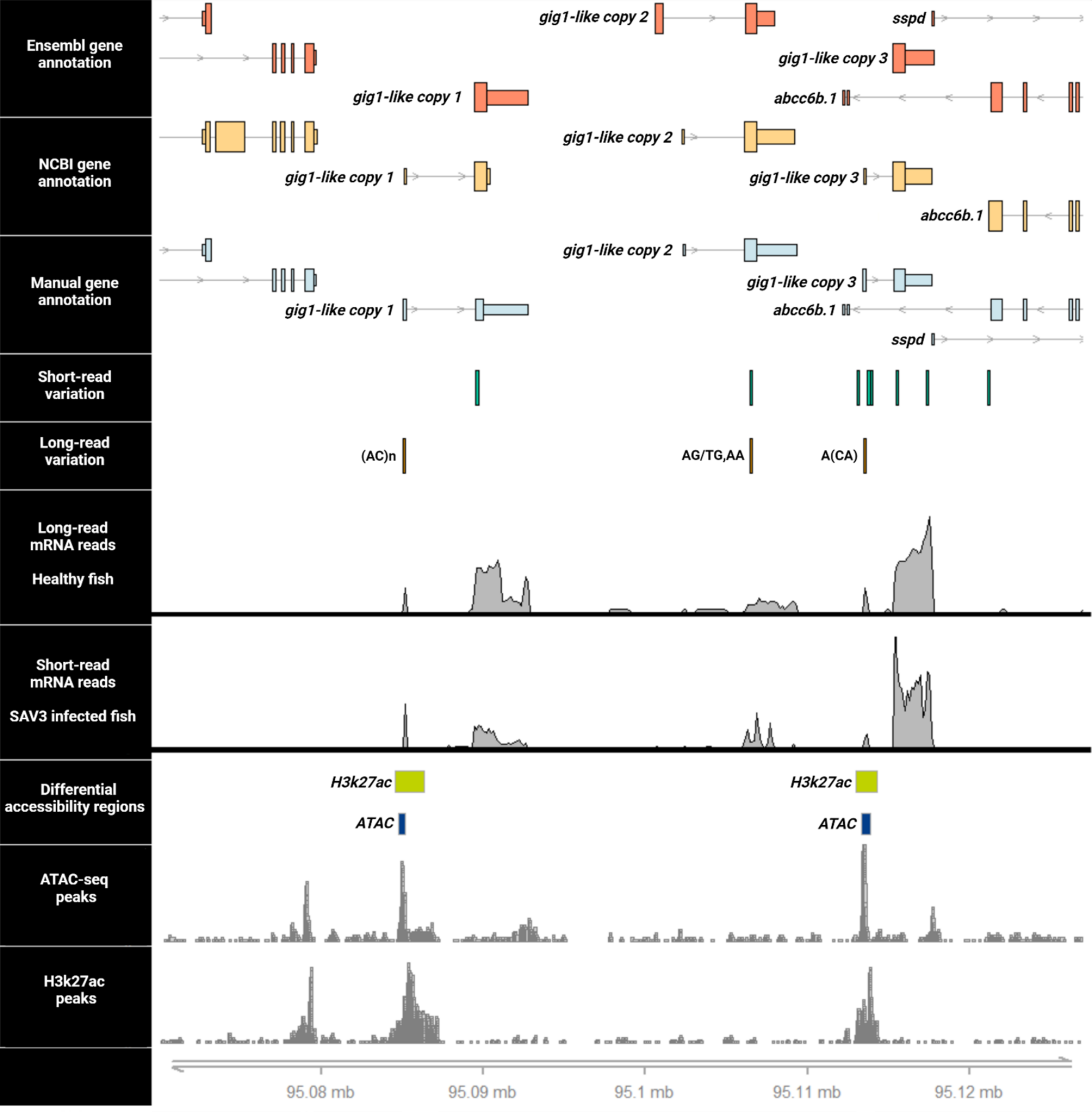
The putative functional variation landscape of the major QTL for PD resistance. From top to bottom, bins represent the gene landscape according to the Ensembl (release 112) and NCBI publicly available gene annotations for Atlantic salmon. Below, the manually curated gene landscape is shown, constructed based on long read and short read sequencing transcriptomics data of healthy and SAV3 infected individuals, respectively. Underneath, genetic variants segregating together with the Ssa03 QTL SNP were detected through short and long read sequencing technologies. Finally, the regulatory landscape of the Ssa03 QTL during interferon (IFN) stimulation was investigated to reveal transcriptionally active regions (ATAC-seq) as well as regions of histone modification (ChIP-seq). Within the first gig1-like copy, an (AC)n microsatellite intersects the coding region of the first exon as well as regions of accessible chromatin and histone acetylation marks, whereas another short variant overlaps the coding region of the second exon, within the 3’ UTR region. One short variant overlaps the coding region of the second gig1-like copy together with a biallelic substitution (AG-> TG, AA). Finally, for the third gig1-like gene copy, five variants located in regions of histone acetylation harboring the gene’s promoter region and five additional variants overlap the coding sequence of the second exon, respectively. Of the variants proximal to the gene copy’s promoter, two SNP variants and another, shorter, microsatellite (ACA<-A) additionally overlap with regions of open chromatin, while only the small deletion intersects with coding sequence

Comparison of the likely candidate genes for PD response between publicly available functional annotations (NCBI and Ensembl genome databases) revealed large structural differences between *gig1-like* gene copies (Fig. 6). In particular, we identified differences in the number of exons for the first and third *gig1-like* gene copies between publicly available annotations as well as differences in the length of the annotated exons for the first and second *gig1-like* genes. In order to address and correct the structural discrepancies of *gig1-like* genes between the NCBI and Ensembl databases for the Ssal_v3.1 assembly, we obtained long-read transcriptome data from the head kidney of 12 healthy fish [20] and combined it with short-read transcriptome data from the SAV3 infectious cohabitation challenge to create a consensus annotation. This approach revealed gene predictions with high structural similarity across the three *gig1-like* genes (Fig. 6) where each gene copy consisted of one small exon (98–136 bp) and one larger exon (2,230–3,310 bp), separated by a long intronic region (Additional file 1: Supplementary Table S3). Open reading frame (ORF) prediction for each *gig1-like* gene detected open reading frames on the second exon of all *gig1-like* genes, coding for proteins of 240, 222 and 222 amino acids for the first, second and third copies, respectively (Additional file 1: Supplementary Table S3).

Following the creation of a consensus annotation of the *gig1-like* genes, we aimed to elucidate the underlying regulatory landscape and identify likely regulators surrounding the *gig1-like* genes. This investigation utilized regulatory information generated using transposase-accessible chromatin assay (ATAC-Seq) as well as chromatin immunoprecipitation assay (ChIP) data from an experiment of IFN stimulation of Atlantic salmon head kidney tissue samples [19]. Mimicking viral infection, samples were treated with 100 µg of poly(I: C) and regulatory signals were compared against those of samples treated with control (PBS media) conditions (Fig. 6). Investigation of regulatory genomic regions within the Ssa03 QTL region revealed both ATAC and H3K27ac peaks overlapping the first exons of the first and third *gig1-like* gene copies, indicating strong promoter activation in these two genes in response to IFN stimulation. In contrast, no such activation was observed for the second *gig1-like* gene copy (Fig. 6).

**Fig. 7:**
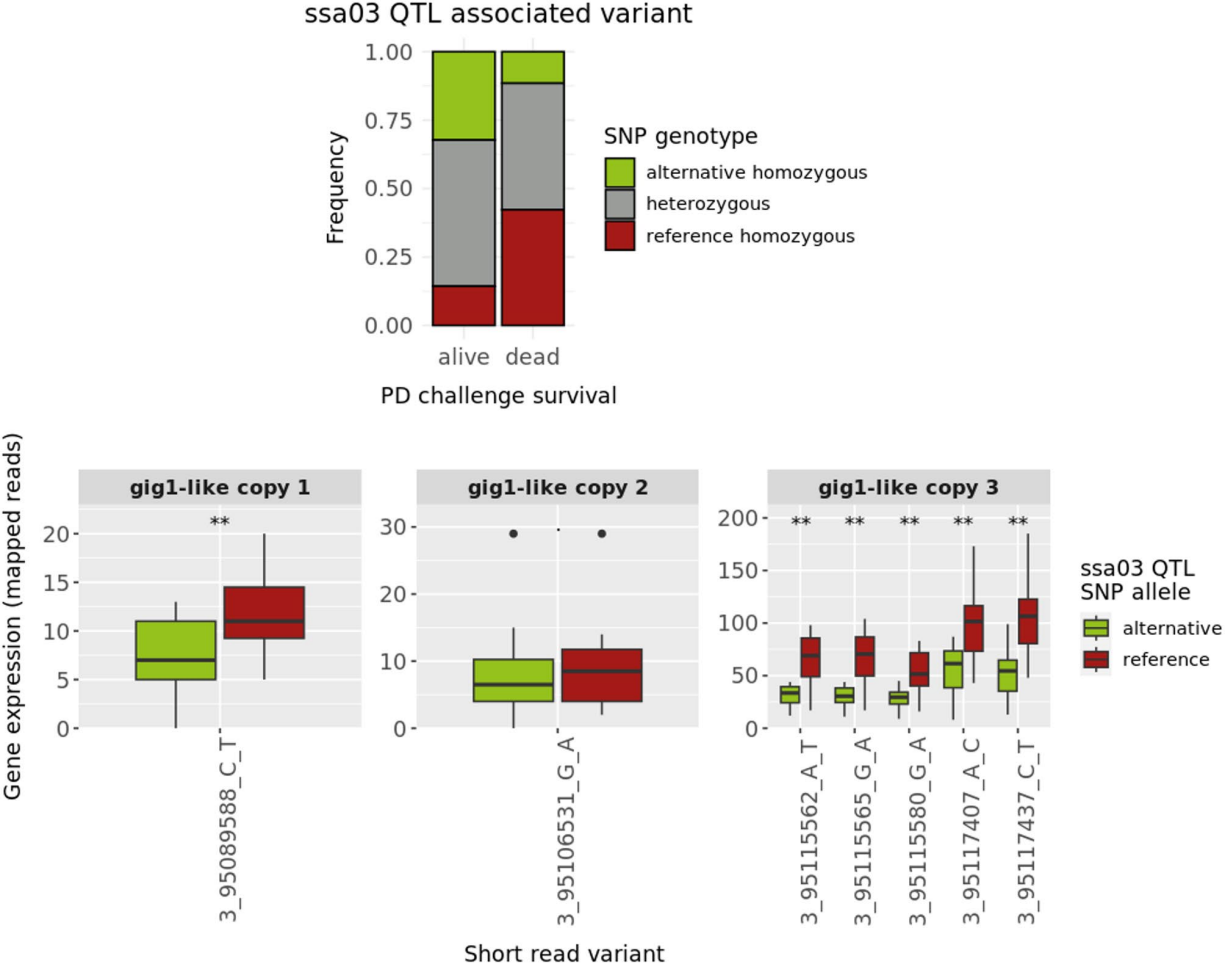
Allele specific expression (ASE) analysis using transcriptomics data from 10 samples from a SAV3 infectious cohabitation challenge. Samples were selected based on their genotype (heterozygous) for the highest disease-associated SNP (top-SNP) within the Ssa03 QTL region. ASE analysis detected 7 biallelic variants whose physical position overlapped transcribed sequences of the three *gig1-like* gene copies and alleles shared high linkage disequilibrium across 293 ancestors of the population used in the PD GWAS study (LD > 0.6). In total, 1, 1 and 5 SNPs overlapped the first, second and third copy of the *gig1-like* gene, respectively. Asterisks across the bottom panels represent variants whose allele phase resulted in significant gene expression differences based on an exact binomial test (p-values < 0.01)

To identify variation overlapping the functional and regulatory landscape within the Ssa03 QTL region we utilized SNPs and short indels called from the aforementioned set of 293 whole-genome short-read sequencing ancestors of the PD challenge individuals. Using these variants, we conducted a linkage disequilibrium (LD) analysis to identify those sharing substantial LD with the Ssa03 top-SNP for PD (r^2^ > 0.6). Out of 115 detected variants only 23 shared substantial LD with the top-SNP (Additional file 1: Supplementary table S4), while four of these overlapped accessible chromatin as well as histone acetylation marks near the promoter region of the third *gig1-like* copy (Fig. 6). Additionally, one, one, and five of the SNPs in linkage phase with the top-SNP were located within the coding regions of the first, second, and third *gig1-like* gene copies, respectively. To identify SNPs potentially affecting *gig1-like* gene expression, we conducted an allele-specific expression (ASE) analysis. This analysis utilized seven SNPs and gene expression data from 10 fish with a heterozygous genotype for the Ssa03 QTL top-SNP. These samples were derived from the SAV3 infectious cohabitation (CH) challenge. The mRNA sequencing reads of heterozygous samples were uniquely mapped to each of the three *gig1-like* genes based on overlapping genetic variation and gene expression was quantified for each SNP allele (see Materials and Methods). The ASE analysis revealed transcriptional activity for all *gig1-like* copies in addition to higher gene expression for SNP alleles in linkage phase with the reference allele of the top-SNP for the QTL on Ssa03, linking higher *gig1-like* gene expression to lower PD resistance (Fig. 7). Notably, significant differences between gene expression level and allele patterns were observed for SNPs within the first and third *gig1-like* gene copies (exact binomial test p-value < 0.01), while no significant gene expression differences were detected for the alleles of the SNP within the second copy. Investigation of the mutational consequences for SNPs with significant allele-specific influence on gene expression revealed three missense mutations for SNPs ssa03_95115562, ssa03_95115565 and ssa03_95115580. overlapping the third *gig1-like* gene. The three mutations resulted in amino acid alterations of three codons, specifically aAg/aTg (K to M amino acid), aGg/aAg (R to K amino acid) and gGt/gAt (G to D amino acid).

### Generating a detailed description of the genomic variation within the Ssa03 QTL region

While short-read sequencing offered valuable insight into the genomic landscape of the Ssa03 QTL in our study, the read lengths of this technology (approx. 120 bp) prohibit the detection and genotyping of large and more complex variation. In order to comprehensively identify all genomic variation underlying the Ssa03 QTL for PD response, including long as well as complex variants overlapping functional and regulatory features, we utilized whole-genome long-read nanopore sequencing data from seven salmon fish that originated from the same aquaculture strain as the GWAS population in this study (AquaGen AS). Variant detection across the seven long-read individuals identified 446 variants within the QTL region (Ssa03: 95,076,090–95,121,178), consisting of 127 SNPs as well as 319 variants of variable complexity including multiallelic variants, microsatellites, tandemly repeated sequence and structural variants (SVs). To assess allele segregation patterns of these variants, disease-associated haplotypes were constructed for the seven fish, classified as either reference (8 haplotypes) or alternative (6 haplotypes) based on the alleles of the 11 SNPs sharing near perfect LD within the Ssa03 haplotype block (Fig. 4). Then, using the SNP with highest association to PD resistance from our GWAS study (top-SNP, ssa03_95086730), we assessed the allele segregation of all variants across the 14 haplotypes, retaining variation in near perfect linkage phase with the reference and alternative top-SNP alleles. Allele segregation analysis identified in total 92 variants including 49 SNPs, 10 insertions, 7 deletions, and 9 microsatellites (Additional file 1: Supplementary table S4). Finally, comparison of the physical position for filtered variants with the functional and regulatory landscape of Ssa03 QTL revealed 25, 8 and 10 variants intersecting the three *gig1-like* gene copies (Fig. 7 and Additional file 1: Supplementary table S4). Notably, a highly variable (AC)n microsatellite intersected the first exon of the first *gig1-like* gene copy in addition to regions of accessible chromatin and histone acetylation marks whereas another shorter microsatellite sequence (ACA/A) overlapped the respective coding and regulatory regions of the third *gig1-like* gene (Fig. 6).

### *Gig1-like* gene phylogenetic analysis

**Fig. 8:**
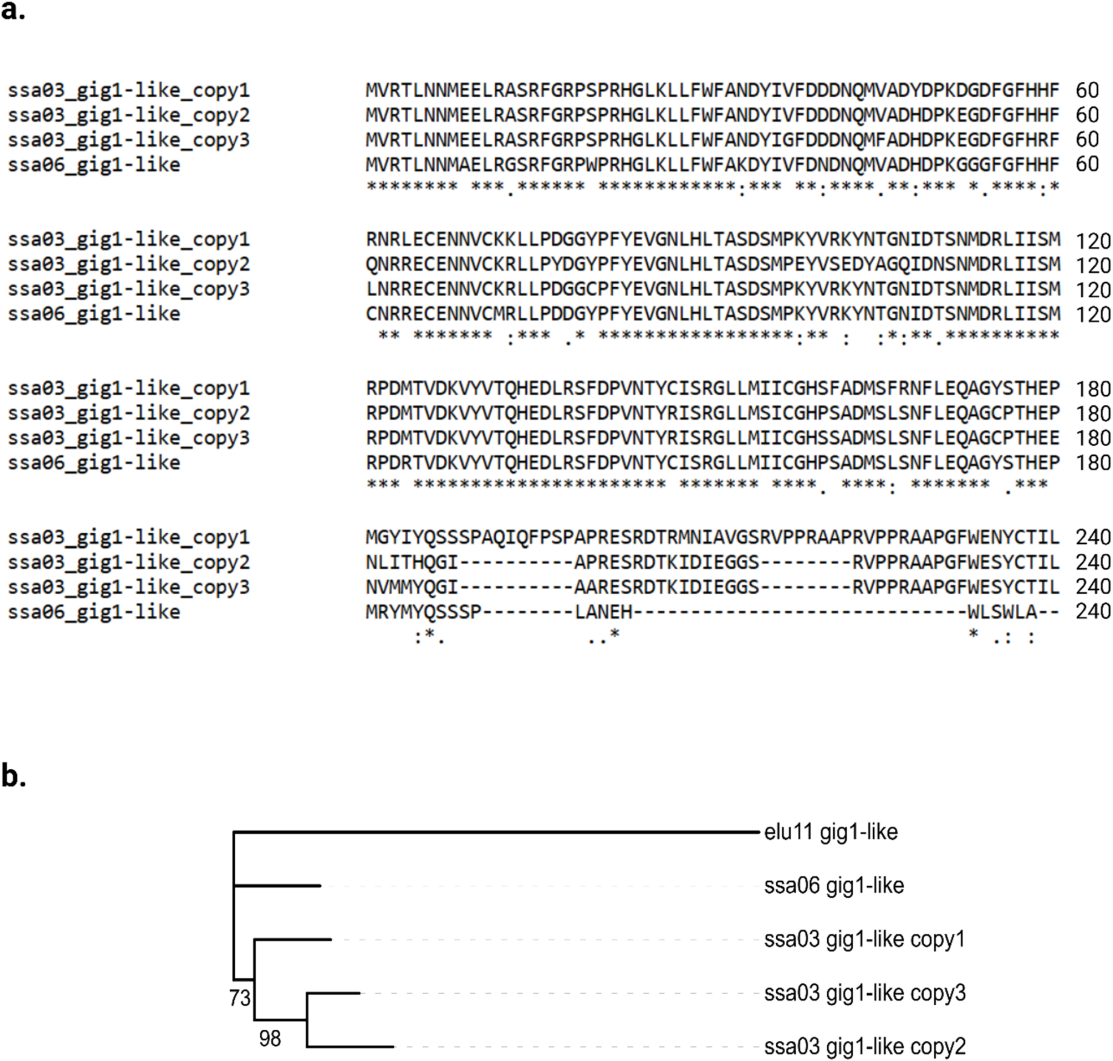
**(a)** Multiple amino acid of the three manually annotated *gig1-like* genes on chromosome 3 (Ssa03) and the *gig1-like* copy located in the ohnologous region on Ssa06. Alignment was carried out using MAFFT v7.511 and default settings. Symbols underneath each peptide represent peptide conservation with asterisk and colon symbols indicating high and moderate positional conservation, respectively. **(b)** Phylogenetic analysis of coding sequence for the Atlantic salmon Ssa03 and Ssa06 *gig1-like* genes. In order to reveal evolutionary relationships across gene paralogs, the gene ortholog of the ancestor species Esox lucius was included as an outgroup (Elu11). The coding sequence of the five genes was aligned using MAFFT v7.511 and a neighbor-joining (NJ) tree was constructed using the gap-free and moderately conserved alignment sites. Numbers on the tree branches indicate the bootstrap support values estimated using 500 replicates

To elucidate the origin of the three *gig1-like* genes on Ssa03, we utilized the protein sequence of the manually annotated *gig1-like* genes together with the protein sequence of a *gig1-like* gene copy located on the ohnolog region of Ssa06 (ENSSSAG00000003892, Ssa06:5,558,971-5,559,951). The amino acid (aa) sequence alignment across the four *gig1-like* genes revealed relatively high similarity among the Ssa03 gene copies, whereas the sequence of the Ssa06 gene was notably shorter, diverging largely between the 191st– 240th aa (Fig. 8a). Focusing on the amino acid sequence alignment of the Ssa03 *gig1-like* genes, two deletions of similar size were identified in the second and third copies compared to the first *gig1-like* gene, in particular one 10 aa and one 8 aa deletion at positions 188 and 214, respectively. To determine whether the observed amino acid differences conferred functional divergence among the Ssa03 gene copies, a functional domain prediction analysis was performed using InterPro [61]. Domain prediction identified the PANTHER domain ‘*SI: CH211-198C19.1-RELATED*’ across all genes (PTHR38706, Additional file 1: Supplementary Table S3), likely indicating the conserved coding region for the *gig1-like* protein. However, no additional domains were identified within the regions showing the largest structural divergence between *gig1-like* gene copies (189–240 aa, Fig. 8a).

Finally, a phylogenetic analysis of the four *gig1-like* genes’ coding sequence was carried out in order to explore the evolutionary history of the Ssa03 copies. In addition to the four salmonid genes, the gene ortholog of *Esox lucius*, a close ancestor species that did not undergo the salmonid-specific whole genome duplication, was included as an outgroup (ENSELUG00000010316, Materials and methods). Phylogenetic analysis indicated that the *gig1-like* gene located on Ssa06 and the first copy on Ssa03 shared higher similarities, whereas the second and third copies on Ssa03 likely arose through segmental duplications of this variant. (Fig. 8b).

## Discussion

Pancreas disease (PD) represents a significant challenge for Atlantic salmon aquaculture as viral outbreaks can have serious implications on animal welfare, production costs, and the reputation of the industry. Understanding the genetic basis of PD resistance is important for developing sustainable and efficient disease management strategies that improve fish welfare, minimize losses, and ensure the long-term sustainability of production. While previous studies have identified several genomic loci associated with PD response, limitations in genomic resources have hindered the fine mapping and thorough investigation of putatively causal variation.

In the present study, we used 938,582 SNPs in order to conduct a high-powered GWAS in a population of fish infected with the SAV3 virus either through peritoneal injection or infectious cohabitation (IP and CH, respectively). Increasing the SNP density of the association study population through genotype imputation allowed us to narrow the Ssa03 and Ssa07 QTL regions from several megabases to 45 and 23 Kb, respectively (Figs. 3 and Additional file 2: Supplementary Fig. 1).

Interestingly, differences in viral transmission (IP vs. CH infection) influenced the association signals of the QTL on Ssa07, limiting the locus’ significance to only IP infected fish (Fig. 3). Peritoneal (IP) infection constitutes an artificial method of viral delivery that stimulates the host’s immune response bypassing any physical immunity barriers [68, 69]. In contrast, during natural PD outbreak scenarios, viral transmission occurs through contact of the fish with water-suspended viral particles released from infected or decomposing cohabitants [4, 70]. In this context, the observed differences between the IP and CH infection groups suggest that varying infection methods may induce distinct immune responses. Supporting this, previous studies examining the impact of different infection models on the response of rainbow trout (*Oncorhynchus mykiss)* to *Yersinia ruckeri* have revealed significant differences in inflammatory (IL-1b1 and IL-8) and anti-microbial (Cath-1 and Cath-2) responses between IP injected fish and infected cohabitants [71]. The use of suboptimal experimental models - and the comparison of results across studies employing different infection methods - can introduce bias into association studies, potentially hindering the detection of disease-resistance loci and, by extension, the discovery of causal variation [72]. Collectively, these findings highlight the importance of selecting representative infection models to accurately characterize and interpret immune responses in aquaculture settings, where infectious disease outbreaks can significantly impact both production efficiency and animal welfare.

Our study focused on the PD associated region on Ssa03, narrowing the position of this QTL to 45 kb (Ssa03: 95,054,413–95,127,543). Based on the Ensembl gene annotation, the region encompasses the genes *nlrp3*,* abcc6.b*,* sspd*, as well as three tandemly duplicated copies of the *gig1-like* gene, suggesting that these genes play important roles in PD resistance. Although several of these genes have been previously reported as likely candidates for PD resistance [10, 14], transcriptomics analysis of fish with quantified SAV3 viral concentration, as well as re-analysis of data from a SAV3 IP survival challenge [15], showed that only the three *gig1-like* genes responded significantly to PD infection within the QTL region (Fig. 4B and C). Consistency in the gene expression findings across studies sheds light on the functional landscape of PD resistance, highlighting *gig1-like* gene copies as the most probable causal candidates.

Grass carp reovirus (*GCRV*)-induced gene 1 (*gig1*) is a fish-specific gene family belonging to interferon (IFN) stimulated genes, that plays an important role in antiviral response [73]. In Atlantic salmon, multiple *gig1* genes have been identified [74], whereas expression of these genes has previously been associated with response to aquaculture diseases, including PD [14, 75, 76]. Although *gig1* has been shown to suppress GCRV viral replication [77, 78], overexpression of *gig1-like* genes in Atlantic salmon is often linked to poor immunity outcomes for virally infected fish [10, 75]. Consistently, our findings showed significantly higher *gig1-like* gene expression in fish with higher SAV3 viral load and greater disease susceptibility (Fig. 5A). Although *gig1-like* genes are induced through an IFN-mediated immune response [73, 74, 78], the observed combination of increased disease susceptibility together with higher gene expression in Atlantic salmon suggests that *gig1-like* genes may serve as indicators of viral replication rather than viral suppression. This points to a potential link between an elevated IFN response and heightened disease susceptibility [76, 79]. IFN stimulation is a key feature of antiviral immunity, however, the negative impact of prolonged IFN response [80] has been previously discussed in the context of aquaculture disease susceptibility [81, 82]. Based on this hypothesis, changes in the transcription of *gig1-like* genes could affect *gig1* effectiveness, leading to a prolonged and ultimately harmful IFN response. Furthermore, the presence of multiple gene copies within the narrow QTL region could introduce additional complexity in *gig1-like* antiviral responses, potentially by altering the regulation of these genes coupled with variations in the transcribed protein [83].

To explore functional differences between the three *gig1-like* gene copies, we used both short and long read sequencing data and confirmed the genes’ structure and protein products (Figs. 5 and 7 and Additional file 1: Supplementary Table S2). Protein sequence alignments of the *gig1-like* genes on Ssa03, together with the gene paralog located on the duplicated region of Ssa06 [84], revealed substantial differences between the *gig1-like* genes across the duplicated regions (Fig. 8). Although the same protein domain was predicted across the four *gig1-like* paralogs, absence of a QTL locus for PD on Ssa06 as well as absence of transcriptomic response suggests that the particular gene copy does not constitute a strong candidate for PD resistance.

Finally, to better understand how the *gig1-like* genes respond to viral infection, we performed an allele specific expression (ASE) analysis using short-read sequencing variation detected in 293 ancestors of the GWAS fish and focused on SNPs located in transcribed regions of the three *gig1-like* gene copies (Fig. 7). Of these, three SNPs represented missense mutations, leading to amino-acid changes within the predicted functional domain of the third *gig1-like* gene copy (peptides 72,73 and 78, Fig. 8a). Location of the SNPs within the gene’s predicted functional domain increase their impact and functional importance, particularly in respect to amino acid changes inside highly conserved peptides (Fig. 8a). However, since *gig1-like* genes are not thoroughly studied yet, the functional consequences of the detected amino acid changes remain elusive.

Moreover, we focused on the regulatory regions within the QTL region for PD and investigated variations with potential functional and regulatory impact on the three *gig1-like* gene copies. Using long-read sequencing information from seven aquaculture fish to construct disease associated haplotypes revealed large and complex variation segregating together with the top-SNP for PD resistance in the QTL region (Additional file 1: Supplementary table S4). Interestingly, an AC(n) microsatellite located on the first *gig1-like* copy, and another shorter microsatellite ACA/A on the third *gig1-like* copy overlapped transcription start sites with regulatory activity during viral infection response (Fig. 6). Comparison of the alleles for these two variants across disease associated haplotypes revealed a link between allele length and disease susceptibility (Additional file 1: Supplementary table S4). Several studies have described the impact of repeat sequence expansion on gene expression [85, 86]. In our study, the overlap between the microsatellite and the promoter region of the first *gig1-like* gene copy, along with additional overlap with regulatory elements, supports the hypothesis that expansions of this particular variant could significantly impact the regulation and expression of the gene. Furthermore, detection of a similar, yet shorter (AC) microsatellite on the third *gig1-like* gene copy could indicate the presence of similar variation occurring across the particular genes. However, the limited number of long-read sequenced individuals in our study restricted further analysis of these variants on *gig1-like* gene expression, underscoring the need for follow up studies to fully understand the mechanisms underlying PD resistance.

As highlighted above, further investigation is needed to validate the functional impact of the three *gig1-like* gene copies and the putative causal variants underlying PD resistance. In our analysis, the high sequence similarity within the PD associated region on Ssa03 posed challenges for short-read sequencing technologies, particularly for detecting complex variants and accurately assigning transcriptomic reads among *gig1-like* gene copies. In this context, targeted long-read sequencing of fish with phenotypic records on PD resistance could provide stronger evidence linking complex genetic variants, such as the polymorphic (AC)n microsatellites, to PD response [87]. Similarly, applying long-read sequencing methods to transcriptomic analyses could yield a more accurate representation of the functional landscape underlying disease resistance [87]. In addition, gene editing approaches, including knock-in or knock-out experiments, as well as gene overexpression experiments in vitro, could be used to validate the functional impact of repeat length expansions in regulation of *gig1-like* gene expression [88]. Furthermore, sequential inactivation of the *gig1-like* copies in SAV3 infected cells could uncover potential regulatory interactions among the three tandem duplicates and shed light on their role in mediating disease response [83]. Investigating the relationship between the *gig1-like* gene expression and PD resistance, along with the effects of the putatively causal variants, could significantly advance our understanding of the molecular mechanisms underlying PD response. Incorporating this knowledge into breeding programs may strengthen disease management strategies, ultimately enhancing animal welfare and promoting the sustainability of aquaculture.

## Conclusions

In conclusion the present study provides a comprehensive analysis of the genomic and functional landscape underlying response to PD, advancing our understanding of the genetic basis of PD resistance. Our study confirmed a major QTL in chromosome Ssa03 and a secondary region on chromosome Ssa07 with the latter locus addressing infection-specific interactions with the host. Transcriptomics analysis of viral load and disease susceptibility within the major QTL for PD resistance on Ssa03 revealed three in-tandem duplicated copies of the *gig1-like* gene as the likely causal gene candidates. Increased expression of these genes in fish with poor disease outcomes suggests that the *gig1-like* copies serve as indicators of SAV3 viral replication levels in infected fish, emphasizing their potential role in aquaculture. Using the latest advances in Atlantic salmon genomics we elucidated the genomic and functional landscape underlying these genes, uncovering variation with putative impact on coding as well as regulatory regions of *gig1-like* genes. Notably, two polymorphic microsatellites overlapped the transcription start of the first and third *gig1-like* copies, respectively, that overlapped transcriptionally active regulatory regions during anti-viral response. Use of long-read sequencing technologies revealed a link between microsatellite allele length and disease susceptibility, underscoring the importance of using advanced genomic technologies to unravel the complex interplay between genetic variation and response to traits with great importance to aquaculture sustainability.

## Electronic supplementary material

Below is the link to the electronic supplementary material.

Supplementary Material 1

Supplementary Material 2

Supplementary Material 3

## Data Availability

The raw genotype, phenotype and whole-genome sequencing data used in this study is property of a commercial enterprise and therefore not publicly available. Reasonable request for access to the raw material should be directed to the corresponding author. Raw material for the analysis of the Atlantic salmon regulatory landscape is available on the European Nucleotide Archive under accession numbers PRJEB50077 and PRJEB56698 for ATAC-seq and ChIP-seq information, respectively. Raw short-read transcriptomics data related to the SAV3 infectious cohabitation challenge is available on the European Nucleotide archive under accession number PRJEB85594. Raw transcriptomics data from the re-analysis of a SAV3 infectious survival challenge are available on the European Nucleotide Archive under accession number PRJNA590166. Additional data supporting the findings of the current study are available upon request to the corresponding author.

## Abbreviations

SAV: Salmonid alphavirus
SAV[1–3]: Subtype 1–7 of the Salmonid Alphavirus
PD: Pancreas disease
IP: Peritoneal injection
CH: Cohabitation
dpi: Days post-infection
QTL: Quantitative trait locus
SNP: Single nucleotide polymorphism
SV: structural variant
GWAS: Genome-wide association study
LD: Linkage disequilibrium
Mb: Mega base-pair
Kb: Kilo base-pair
TPM: Transcripts per million mapped reads
WGS: whole genome sequence
GRM: Genomic relationship matrix
PC: Principal component
qPCR: quantitative Polymerase chain reaction
ONT: Oxford Nanopore Technologies
FDR: False discovery rate
DEG: Differential gene expression
ASE: allele-specific expression
ORF: Open reading frame
IFN: Interferon
HK: head kidney
GCRV: Grass carp reovirus
nlrp3: NACHT, LRR and PYD domains-containing protein 3-like
gig1: Grass carp reovirus-induced gene 1
abcc6b.1: ATP-binding cassette, sub-family C, member 6b, tandem duplicate 1
sspd: salmo salar pancreas disease
slc38a2: solute carrier family 38 member 2
scaff11: SR-related CTD-associated factor 11
mRNA: messenger ribonucleic acid
DNA: Deoxyribonucleic Acid
aa: amino acid
