# Supplementary figures and images for: Dissecting the genetic basis of response to salmonid alphavirus in Atlantic salmon
Source: BMC Genomics. 2025 Jul 11;26:657. doi: 10.1186/s12864-025-11735-2 (PMC12247413; doi:10.1186/s12864-025-11735-2)

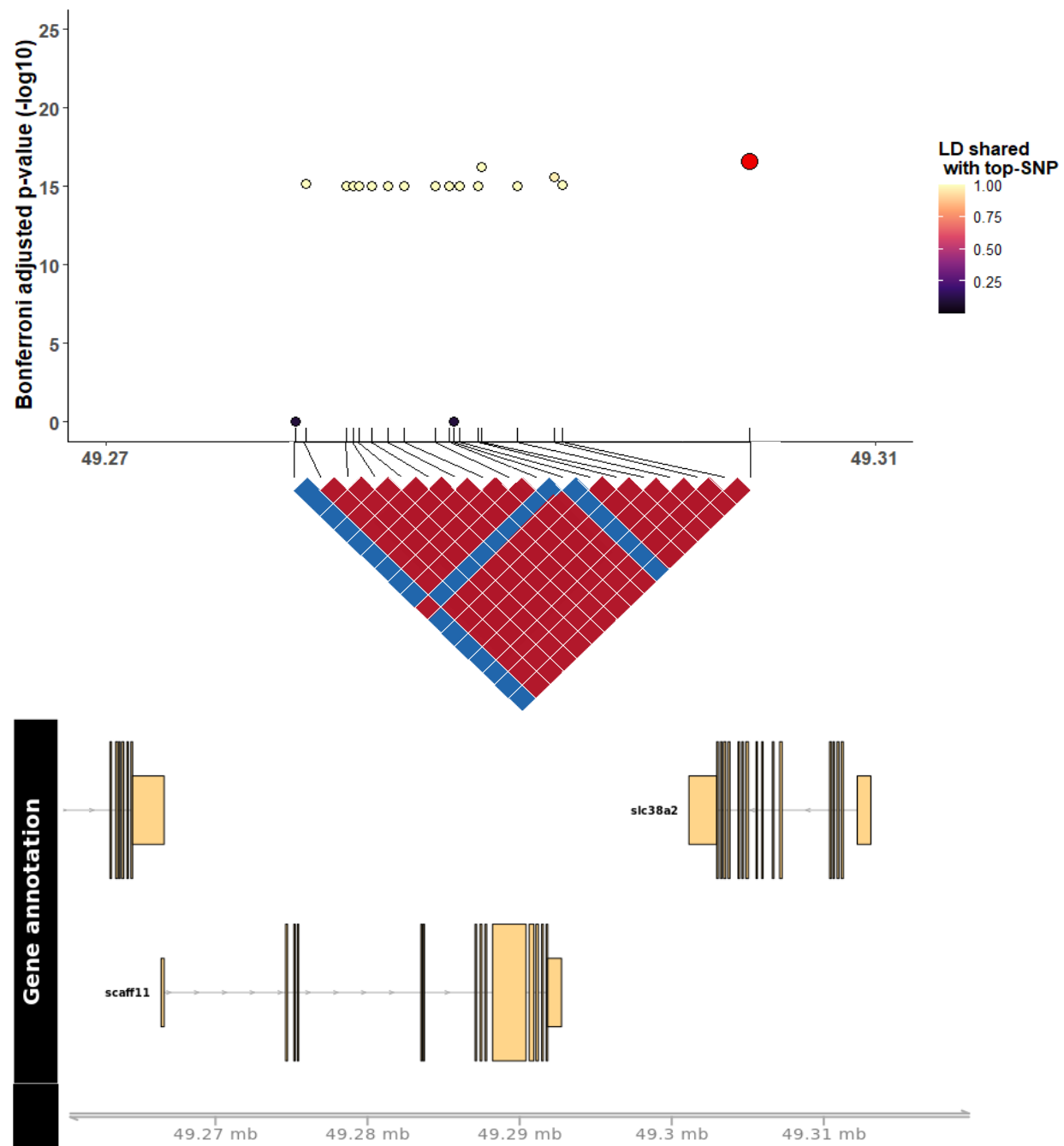

**Supplementary Figure 1**

Supplement: Supplementary file 2 — Supplementary Material 2 [file 12864_2025_11735_MOESM2_ESM.pdf]
