# Supplementary material for: Dissecting the genetic basis of response to salmonid alphavirus in Atlantic salmon
Source: BMC Genomics. 2025 Jul 11;26:657. doi: 10.1186/s12864-025-11735-2 (PMC12247413; doi:10.1186/s12864-025-11735-2)
Supplement: Supplementary file 3 — Supplementary Material 3 [file 12864_2025_11735_MOESM3_ESM.pdf]

Supplementary information

File name: Additional file 1

File format: Excel worksheet (.xlsx)

Titles and description of file components:

Supplementary table S1 Estimated heritability and genetic correlation for PD resistance (death or survival) and survivability (survived days post infection) of fish infected with Salmonid Alphavirus (SAV3). In total 5,628 individuals hatched between the years 2018-2020 participated in three independent infectious challenges either by intraperitoneal SAV3 injection (IP group: 1,112, 1,023 and 1,060 fish of year class 2018, 2019 and 2020 respectively) or by infectious cohabitation with virally injected individuals (CH group: 1,137 and 1,296 fish of year class 2018 and 2019, respectively). Heritability and genetic correlations were estimated separately for each year-class and collectively based on survival as well as infection group using a bivariate model (see Materials and methods).

Supplementary table S2 Haplotype frequency for SNPs sharing near perfect linkage disequilibrium (LD) with the highest PD associated SNPs on chromosomes Ssa03 and Ssa07. Two narrow haplotype blocks were identified on chromosomes Ssa03 and Ssa07 that included the highest associated SNPs for PD resistance, respectively (referred to as top-SNPs). Within each haplotype block, 11 and 16 SNPs shared near perfect LD (r^2 > 95%) with the Ssa03 and Ssa07 top-SNP, respectively (column 1). Haplotype combinations for these SNPs across the imputed SAV3 challenge population are shown on the second column of the table. The frequency of each haplotype within the population is shown in columns 3 and 4. Out of the 16 detected haplotypes, only four haplotypes were found in more than 20% of the total population, highlighted in this table.

Supplementary table S3 Manual annotation of the three *gig1-like* candidate genes on the Ssa03 QTL region for PD resistance. The distribution of mRNA sequence reads from long read sequencing transcriptomics data from healthy fish was compared against short-read information from SAV3 infected fish in order to validate the structural similarity between the three in tandem duplicated copies of the *gig1-like* gene. The second and third column in the table show the physical coordinates of the exons identified for each copy of the *gig1-like* gene based on the visualized distribution of transcriptomics information. The physical position of detected open reading frames (ORF) and amino acid length for the second exon of each gene copy are shown in columns 8 through 10 while the complete amino acid sequence of the same reading frames is provided in rows 8 through 27. Finally, the results of functional domain prediction analysis with InterPro [60] and the length of the predicted functional domains are shown in columns 11 and 12, respectively.

Supplementary table S4 Integration of short and long read sequencing elucidates the genomic landscape of PD resistance. Simple and complex variants were identified within the PD associated haplotype block on Ssa03 using data from 293 whole-genome short-read resequencing ancestors of the SAV3 population and from 14 disease-associated haplotypes constructed from long-read sequencing data from seven fish of the same aquaculture strain (AquaGen AS). The table includes variants sharing substantial LD across the 293 ancestors ($r^2 > 0.6$) and variants with near perfect allele segregation patterns across disease-associated haplotypes for the reference or alternative Ssa03 top-SNP allele. Columns 1-4 show the physical coordinates and respective reference and alternative alleles for each variant. For multi-allelic variants, multiple alternative alleles are separated by commas. The putative functional impact of variants is shown in column 5, estimated based on functional and regulatory (ATAC-seq and ChIP-seq) information. In column 6, the detection method of each variant is noted,

classified as either short or long read sequencing or combination of methods. For variants detected via long-read sequencing, columns 7-20 show the variants' haplotype allele across the disease associated haplotypes. For each haplotype, 0 indicates the reference allele haplotype while any other number indicates the respective alternative allele for the same variant.

File name: Additional File 2

File format: PDF (.pdf)

Title and description of file components:

Supplementary Figure 1 Linkage disequilibrium and haplotype block analyses within the Ssa07 QTL region. Each colored point represents an individual SNP within the QTL region for PD (x-axis) with statistical association for SAV3 survival (y-axis), while the large red circle indicates the SNP with highest association to PD resistance, hereby referred to as top-SNP for the respective QTL. The color for each point represents linkage disequilibrium (LD) shared between the respective SNP and the topSNP of the Ssa07 QTL, while the LD plot below highligths a narrow genomic region of high LD. Underneath, the functional landscape overlapping the QTL region is shown, based on data obtained from the Ssal_v3.1 annotation in theEnsembl genome database (release 112). In particular, the narrow QTL region overlapped two genes, namely scaff11 and slc38a2
